# Wide Distribution and Genetic Diversity of *Babesia microti* in Small Mammals from Yunnan Province, Southwestern China

**DOI:** 10.1371/journal.pntd.0005898

**Published:** 2017-10-23

**Authors:** Zi-Hou Gao, Tao-Hua Huang, Bao-Gui Jiang, Na Jia, Zheng-Xiang Liu, Zong-Ti Shao, Rui-Ruo Jiang, Hong-Bo Liu, Ran Wei, Yu-Qiong Li, Hong-Wu Yao, Michael E. von Fricken, Jia-Fu Jiang, Chun-Hong Du, Wu-Chun Cao

**Affiliations:** 1 Yunnan Institute of Endemic Diseases Control and Prevention, Yunnan, P.R., China; 2 State Key Laboratory of Pathogen and Biosecurity, Beijing Institute of Microbiology and Epidemiology, Beijing, P. R., China; 3 Chinese PLA General Hospital (301 Hospital), Beijing, P.R., China; 4 George Mason University, Dept. of Global and Community Health, Fairfax, VA, United States of America; 5 Duke University, Duke Medicine, Division of Infectious Disease, Durham N.C., United States of America; University of California San Diego School of Medicine, UNITED STATES

## Abstract

**Background:**

*Babesia*, usually found in wild and domestic mammals worldwide, have recently been responsible for emerging malaria-like zoonosis in infected patients. Human *B*. *microti* infection has been identified in China, primarily in the Southwest along the Myanmar border but little direct surveillance of *B*. *microti* infection in rodents has been carried out here (Yunnan province). In this region, a diverse topographic range combined with tropical moisture sustains a high biodiversity of small mammals, which might play important role on *Babesia* transmission.

**Methods:**

Small mammals were captured in 141 sample locations from 18 counties located Yunnan Province, and screened for *B*. *microti*-like parasites infection by a nested PCR to target 18S rRNA gene of *Babesia*, plus directly sequencing for positive samples. Univariate and multivariate forward stepwise logistic regression analysis was used to access the association between infections and some related risk factors.

**Results:**

Infection with *Babesia microti* was confirmed in 2.4% (53/ 2204) of small mammals. Significant differences in prevalence rates of *B*. *microti* were observed based on variations in forest, agricultural, and residential landscapes. Furthermore, adult small mammals had higher prevalence rates than younger, pubertal mammals. The near full-length 18S rRNA gene revealed that there were two types of *B*. *microti*, Kobe and Otsu, which demonstrate the genetic diversity and regional distribution.

**Conclusions:**

There exists a wide distribution and genetic diversity of endemic *B*. *microti* in Southwestern China, warranting further investigations and monitoring of clinical disease in individuals presenting with *Babesia* like symptoms in these areas.

## Author summary

*Babesia* spp are garnering more attention as causative agents of human disease, with *B*. *microti* responsible for most cases globally. Our study documents potential small mammal reservoir hosts, collected from a large of sample sites, with PCR and sequencing identifying the wide distribution and genetic diversity of endemic *B*. *microti* in Southwestern China. Our study adds to body of literature on *Babesia* in China, while focusing on potential rodent reservoirs of disease. This report will be important for public health, and for researchers in the field of parasitology.

## Introduction

Intraerythrocytic protozoan parasites from the genus *Babesia*, found in wild and domestic mammals worldwide, have recently been responsible for emerging malaria-like zoonosis in infected patients. Transmitted by ticks, causative agents of human babesiosis vary by geographic region, with *Babesia microti*, maintained in rodent reservoirs, causing disease in the United States, whereas in Europe, the bovine pathogen *B divergens* is responsible for reported human cases [1]. Only a few human cases of babesiosis have been reported in Asia over the past two decades [2–6]. Recently, 61 confirmed cases of babesiosis have been reported in China; ten patients infected with *B microti* mainly in the Yunnan Province, two with *B*. *divergens* in Shandong Province, and 49 with *B*. *venatorum* (formerly called Babesia sp. EU1) in northern China [7–9]. There have been other reports of suspected *Babesia-*like cases, including the first reported case in the Yunnan Province, however they were not clearly identified for exact species [10]. *B*. *microti* has been identified in small wild rodents found in North America, Europe, and Japan [11], and occasionally in eastern China [12]. In southwestern China, the Yunnan province’s topographic range combined with tropical moisture, sustains a high biodiversity of small mammals that could potentially maintain *Babesia spp*. However, little direct surveillance on rodents has been conducted in this area, despite 16% (10/61) of recent reported cases of Babesiosis occurring here. This research aims to investigate the role small mammal play in *Babesia* transmission in the Yunnan province.

## Materials and methods

### Ethics statement

The research protocol involving with trapping wild small animals and collecting samples was approved by the Animal Subjects Research Review Boards at the Yunnan Institute of Endemic Diseases Control and Prevention (2013–003), in accordance with the medical research regulations of China and the Regulation of the People’s Republic of China for the Implementation of the Protection of Terrestrial Wildlife.

### Collection of small mammal samples

During May 2011 to November 2015, small mammals were captured using animal snap traps set at agricultural, forest and residential landscapes in 141 sample locations from 18 counties located Yunnan Province (Table 1), with a total of 200 snap traps per sample location were placed for three consecutive nights. Mammal species was identified according to external morphology, fur color, measurements and visible characters of dentition [13], with recordings of sex, developmental stage, and environment taken at the time of sample processing. After identification of species, spleen tissues were removed from the animals and stored in liquid nitrogen until tested. For unidentified species in the field, the craniums were brought to the laboratory for further identification.

**Table 1:** Prevalence of *B*. *microti* in small mammals from different survey sites.

Counties	No. of tested	No. of positive for *B*. *microti* (%)	Latitude	Longitude
Tengchong	39	11(28.21)	24.92	98.73
Lushui	114	22 (19.30)	26.46	99.65
Jinggu	76	3 (3.95)	23.58	100.68
Shiping	128	3 (2.34)	23.79	102.36
Weixi	99	2 (2.02)	27.31	99.28
Menghai	175	3 (1.71)	22.02	100.49
Yongde	215	3 (1.40)	24.12	99.40
Mengla	81	1 (1.23)	21.49	101.58
Deqin	346	4 (1.16)	28.08	99.20
Shangri-La	91	1 (1.10)	28.45	99.80
Yulong	224	0 (0)	26.96	100.31
Gongshan	100	0 (0)	28.09	98.67
Fugong	134	0 (0)	26.60	98.97
Yunxian	68	0 (0)	24.73	100.34
Ninger	88	0 (0)	23.06	101.05
Yiliang	94	0 (0)	24.91	103.18
Mile	110	0(0)	24.09	103.41
Mengzi	22	0(0)	23.46	103.37
Total	2204	53 (2.40)		

### DNA extraction and pcr analysis

DNA was extracted from spleen tissue using the DNA blood and tissue kit (Tiangen Biotechnique, Beijing, China) according to the manufacturer’s instructions. A nested PCR to target partial sequences within the 18S rRNA gene of *B*. *microti*-like parasites was done as previously described by Tsuji et al [11]. For *B*. *microti* PCR-positive samples, the nearly complete 18S rRNA gene sequence was amplified using primer pairs of Piro0F and Piro6R and of Piro1F and Piro6R designed by Kawabuchi et al [14]. Amplicons were directly sequenced with automated DNA sequence (ABI PRISM 373; Perkin-Elmer, Norwalk, CT). Sequences analysis was carried out using CLC and a FASTA search on the Genbank database. A Phylogenetic tree was constructed using MEGA software (version 6.06) [15].

### Statistical analysis

Univariate analyses was used to access the association between rodents species, gender, developmental stage, sampling location, environment landscape as well as altitude and *B*. *microti* by using chi-square test or a Fisher’s exact test. All variables with a *P*-value of <0.05 from univariate analysis were entered into a multivariate forward stepwise logistic regression analysis. All analyses were conducted using SPSS (version 17.0, SPSS Inc. Chicago, IL).

## Results

A total of 2204 small mammals belonging to 50 species, 26 genera and 9 families from four orders were collected at 141 sample locations across 18 counties of the Yunnan Province. (Table 1, Table 2). *Rattus tanezumi* made up 23.5% (n = 518) of all trapped small mammals. Fifty-three small mammals from 12 species, primarily *Rattus brunneusculus* (7.53%, 7/93), *Apodemus draco* (5.91%, 13/220), *Eothenomys eleusis* (14.30%, 5/35) and *Rattus tanezumi* (2.70%, 14/518), were actively infected with *B*. *microti* (Table 1). Positive rodents originated from ten counties including Tengchong, Lushui, Jinggu, Shiping, Weixi, Menghai, Yongde, Mengla, Deqin and Shangri-La (Table 1, Table 2). The prevalence of *B*. *microti* in small mammals in agricultural landscape, forest landscape and residential landscape were 1.79%, 3.37% and 0.93% respectively with significant difference (p = 0.043) (Table 3). There was no significant difference in prevalence of *B*. *microti* between male and female small mammals (χ2 = 0.022, p = 0.882, Table 3). However, the prevalence of *B*. *microti* in adult small mammals (2.69%) was significantly higher (χ2 = 5.486, p = 0.019) than that in the pubertal mammals (0.37%), as depicted in Table 3. The prevalence of *B*. *microti* in small mammals at the altitude classes of <1500 meters, 1500–2500 meters, >2500 meters were 2.76%, 5.04% and 0.77%, respectively (Table 3). The multivariate logistic regression analysis revealed that sampling at 1500–2500 meters, adult life stage, and forest landscape were risk factors associated with infection by *B*. *microti* (Table 4).

**Table 2:** Prevalence of *B*. *microti* in small mammals of different species.

Orders	Families	Genera	Species	No. of tested	No. of positive (%)
Rodentia	Muridae	*Rattus*	*R*. *brunneusculus*	93	7(7.53)
			*R*. *tanezumi*	518	14(2.70)
			*R*. *nitidus*	42	0(0)
			*R*. *norvegicus*	16	0(0)
			*R*. *turkestanicus*	7	0(0)
		*Apodemus*	*A*. *draco*	220	13(5.91)
			*A*. *latronum*	90	1(1.11)
			*A*.*chevrieri*	184	0(0)
		*Mus*	*M*. *pahari*	84	1(1.19)
			*M*. *caroli*	75	0(0)
			*M*. *musculus*	12	0(0)
		*Niviventer*	*N*. *confucianus*	108	1(0.93)
			*N*. *eha*	8	0(0)
			*N*. *fulvescens*	9	0(0)
			*N*. *andersoni*	24	0(0)
		*Vernaya*	*V*. *fulva*	2	0(0)
		*Micromys*	*M*.*minutus*	1	0(0)
		Bandicota	*B*. *indica*	2	0(0)
		Berylmys	*B*. *bowersi*	7	0(0)
		*Leopoldamys*	*L*. *edwardsi*	4	0(0)
	Cricetidae	*Eothenomys*	*E*. *eleusis*	35	5(14.30)
			*E*.*custos*	65	2(3.08)
			*E*.*miletus*	88	0(0)
			*E*. *olitor*	4	0(0)
			*E*.*cachinus*	9	0(0)
			*E*. *proditor*	8	0(0)
		*Pitymys*	*P*.*leucurus*	35	1
		*Volemys*	*V*. *clarkei*	13	0(0)
	Sciuridae	Dremomys	*D*. *pernyi*	20	0(0)
		*Tamiops*	*T*.*swinhoei*	3	0(0)
	Dipodidae	*Eozapus*	*E*.*setchuanus*	2	0(0)
Insectivora	Soricidae	*Crocidura*	*C*. *russula*	18	1(5.56)
			*C*. *attenuate*	40	0(0)
			*C*. *dracula*	58	0(0)
			*C*.*horsfieldi*	4	0(0)
			*C*.*lasiura*	1	0(0)
		*Soriculus*	*S*. *leucops*	25	0(0)
			*S*. *nigrescens*	7	0(0)
		*Sorex*	*S*. *alpinus*	11	0(0)
			*S*. *unguiculatus*	5	0(0)
			*S*.*cylindricauda*	15	0(0)
		*Suneus*	*S*. *murinus*	54	1(1.85)
		*Anourosorex*	*A*. *squamipes*	48	0(0)
	Erinaceidae	*Neotetracus*	*N*. *sinensis*	15	6(40.00)
		*Hylomys*	*H*. *suillus*	14	0(0)
	Talpidae	*Scaptonyx*	*S*. *fusicaudus*	3	0(0)
		*Nasillus*	*N*. *gracilis*	13	0(0)
Lagomorpha	Ochotonidae	*Ochotona*	*O*. *thibetana*	45	0(0)
			*O*. *gloveri*	3	0(0)
Scandentia	Tupaiidae	*Tupaia*	*T*.*belangeri*	37	0(0)

**Table 3:** Risk factors related to *B*. *microti* based on univariate analyses.

Variable	Simple size		*Babesia microti* infection		
	cases	constituent ratio(%)	positive rate	χ2	*p*
altitude(m)					
~1500	833	37.79	2.76		
1500~2500	456	20.69	5.04	24.466	<0.01
2500~	915	41.52	0.77		
gender					
male	1059	48.05	2.46	0.022	0.882
female	1145	51.95	2.36		
age					
adult	1932	87.66	2.69	5.486	0.019
pubertal	272	12.34	0.37		
landscape					
agricultural	1176	53.36	1.79		
forest	920	41.74	3.37		0.043^a^
residential	108	4.9	0.93		

a) Fisher’s exact test

**Table 4:** Risk factors related to *B*. *microti* based on multivariate logistic regression.

Variable	OR (95% CI)	*p*
altitude(m)		
2500~	1	
1500~2500	10.286(4.334~24.409)	<0.01
~1500	7.660(3.090~18.989)	<0.01
age	0.125 (0.017~0.917)	0.041
landscape		
agricultural	1	
forest	3.180(1.715~5.896)	<0.01
residential	0.446(0.059–3.364)	0.434

**Fig 1:**
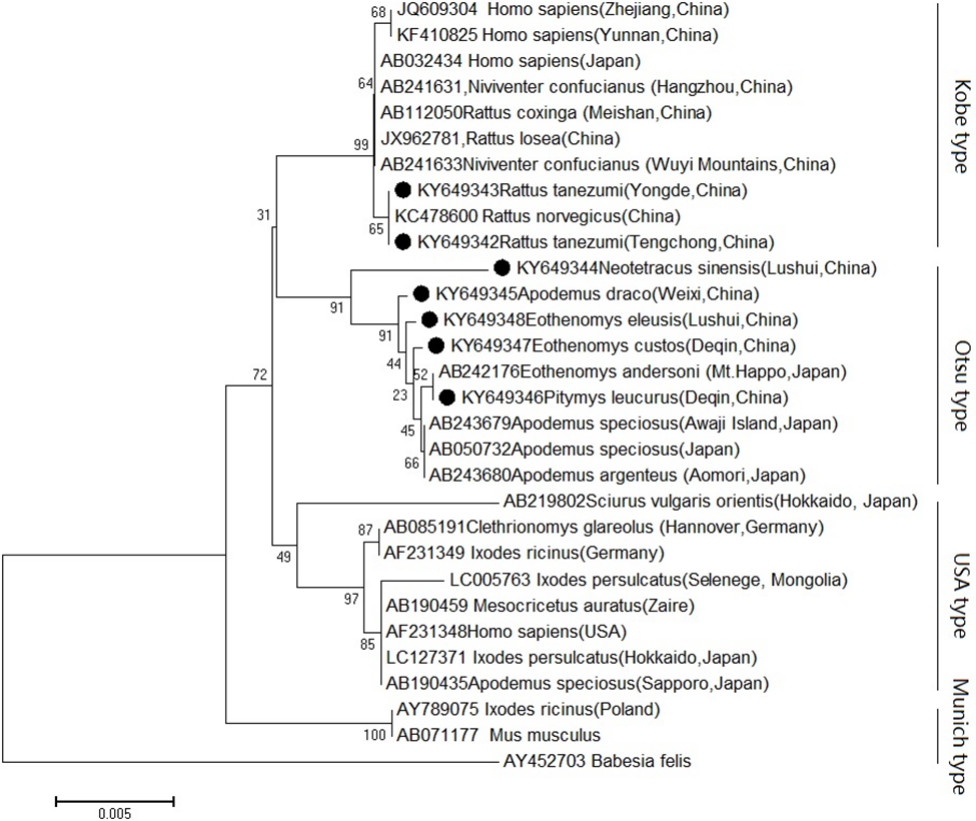
Neighbor-joining phylogenetic tree based on a comparison of *Babesia microti* 18S rRNA gene sequences obtained from Yunnan small mammals with *Babesia microti* reference strains. *Babesia felis* was included as the outgroup. The number on each branch shows the percent occurrence in 1,000 bootstrap replicates. Black circles stood for novel sequences identified in this study.

Near full-length 18S rRNA gene sequences were recovered from the 21 out of 53 *B*. *microti-*positive samples. The sequence from four *Neotetracus sinensis*, which were collected from Lushui County (C7), were distinguished those from four *Eothenomys eleusis* collected at the same location. While the sequences from three *R*. *tanezumi* and three *R*. *brunneusculus* collected at Tengchong County were identical. Phylogenetic analyses based on different representative sequences (deposited in GenBank with accession nos. AY649342-AY649348) revealed that they were divided into two types of *B*. *microti*, with two belonging to Kobe type, and five belonging to Otsu type (Fig 1).

## Discussion

Our present study shows the wide distribution and genetic diversity of *Babesia microti* in small mammals captured from the Yunnan Province based on the large number of samples tested and extent of geographic coverage. More than 100 Babesia species may infect a variety of both wild and domestic animals, but few have been confirmed to infect people, among which *B*. *microti* is the main species globally that causes human babesiosis [1]. *B*. *microti* infection was found in field rodents sampled in proximity to cases of human babesiosis in Taiwan and Zhejiang Province, China [12, 16]. However, until now, *Babesia* has not been found in small mammals from Yunnan Province. The prevalence of *B*. *microti* in small mammals in our study [2.40%) was in line with the low prevalence reported in the Dapan Mountains of Zhejiang Province (1.30%) [12]. However, the *Rattus tanezumi* and *Nivivnter confucianus* in Dapan Mountain had a much higher prevalence (5.6% and 20% respectively) than that of the same two species sampled in our study (2.70% and 0.93%). In China, there were four provinces (Zhejiang, Fujian, Beijing and Yunnan Provinces) which reported the *B*. *microti* infection in *Nivivnter confucianus* [12, 17, 18], which may be the main reservoir hosts in China. Our study found 12 species were positive for *B*. *microti*, which suggests a possible variety of reservoir hosts in the Yunnan Province.

The spleen is a secondary lymphoid organ specialized in filtering blood-borne pathogens and the splenic red pulp contains macrophages that trap and remove damaged red blood cells, which is good choice for diagnosis of Babesia infection [19]. The nested PCR is the method of choice for diagnosis of *B*. *microti* in the blood of rodent hosts and has a higher sensitivity than nested those for spleens [20]. However, in present research, all small mammals were collected by snap traps and died before we can get their blood. These are some limitations of our project.

The survey sites contained a broad range of altitudes from 500 meters to 4500 meters. Among the three altitude classes, small mammals with highest prevalence of *B*. *microti* distributed between 1500~2500 m. This result may be due to the temperature and humidity at this altitude range, which is conducive for tick growth and reproduction. Our findings suggests that the prevalence of *B*. *microti* in small mammals varies by landscape type, which is likely related to tick vector density and preferred habitat, with prevalence rates as from highest to lowest as follows; forest landscape > agricultural landscape > residential landscape. This study reiterates the need for individuals traveling into potential tick habitats, like the forest, to take proper protective measures to limit tick bite exposure. The prevalence of *B*. *microti* in adult small mammals was significantly higher than pubertal ones, which is likely due to dynamics of parasite carriage and the influence of age on likelihood of exposure.

This research found a high prevalence of *B*. *microti* in small mammals in Lushui County (Table 1) with different gene-types carried by different host species. Notably, the prevalence of *B*. *microti* in Tengchong (Table 1) was the highest among the survey sites, where six near full-length 18S rRNA gene identical sequences of *B*. *microti* were recovered from house rats, *R*. *tanezumi* and *R*. *brunneusculus*, which shared 99.9% identity with a human case of babesiosis reported from that region (GenBank KF410825). These findings suggest that there may be an important natural foci of *B*. *microti* in Tengchong and adjoining Lushui County. The ecology of *B*. *microti* in sampled wild animals from Yunnan province, suggests that *Babesia* infection in humans, livestock and ticks warrants further investigation.

## Supporting information

S1 Data The original data underlying the findings described in the manuscript. (XLSX)
